# Impact of Patient Age on Age-Adjusted Minimum Alveolar Concentration Fractions and Prolonged Tracheal Extubations Where Anesthesia Machines Display the Fractions

**DOI:** 10.7759/cureus.111319

**Published:** 2026-06-22

**Authors:** Franklin Dexter, Anil A Marian, Richard H Epstein

**Affiliations:** 1 Anesthesia, University of Iowa, Iowa City, USA; 2 Anesthesiology, University of Miami Miller School of Medicine, Miami, USA

**Keywords:** airway extubation, delayed emergence from anesthesia, inhalational anesthetics, managerial epidemiology, operating rooms, organizational efficiency, retrospective studies, time factors

## Abstract

Introduction

Prolonged time to tracheal extubation after general anesthesia is defined as an interval of 15 minutes or longer from the recorded end of surgery to the actual removal of the tracheal tube. Thereafter, the operating room team is idle, so prolonged extubations reduce workflow and the productivity of anesthesia clinicians. During inhalational anesthesia, the overwhelming majority of prolonged times to tracheal extubation are caused by substantial residual doses at the completion of the surgical procedure. The minimum alveolar concentration (MAC) fraction is the end-tidal inhalational agent concentration divided by the agent’s altitude- and age-adjusted minimum alveolar concentration for a 40-year-old. Age adjustment is essential because volatile anesthetic requirements decline markedly with increasing age, meaning that a fixed percentage concentration represents a much higher effective dose in an elderly patient than in a young adult. Previously, the authors showed a substantial positive correlation between the MAC fraction at the end of surgery and prolonged extubations. In the current study, we tested three hypotheses on the associations between MAC fraction for maintenance of anesthesia and patient age, MAC fraction at the end of surgery and patient age, and prolonged extubations and patient age.

Methods

Our retrospective cohort study used a subset of the same dataset from the University of Iowa that was used in an earlier investigation of inhalational agent dosing behaviors. The anesthesia machines displayed MAC fractions. The cohort of *n* = 75,050 cases had general anesthesia, tracheal intubation, a mean MAC fraction > 0.6 throughout the case, and patient age > 30 years. The associations between patient age and MAC fractions were evaluated using Spearman's rank correlations for comparison with research done at another institution. The associations between prolonged extubations and MAC fractions were tested using the Wilcoxon-Mann-Whitney test.

Results

With care provided using anesthesia machines that display MAC fractions, there was a positive correlation (*p* < 0.0001) between age and MAC fraction, but an association of tiny magnitude: Spearman's rank correlation 0.021, 95% confidence interval 0.014 to 0.028. The correlation was significantly lower than the previously published rank correlation of 0.12 when machines lacked the capability to display MAC fraction (*p* < 0.0001). There was no significant association between age and the MAC fraction at the end of surgery (rank correlation: 0.006; 95% confidence interval: -0.001 to 0.013; *p* = 0.105). There was no significant association between age and prolonged extubation (area under the receiver operating characteristic curve of 0.503; *p* = 0.28).

Conclusions

Prolonged times to tracheal extubation are economically important. Our findings demonstrate that when using anesthesia workstations equipped with real-time MAC fraction displays, the confounding effect of patient age on anesthetic maintenance dosing was effectively neutralized. At the completion of surgery, there was no residual correlation between patient age and the remaining depth of anesthesia. Consequently, the incidence of prolonged extubation was independent of patient age, confirming that most delays in emergence were driven by clinician titration behaviors rather than patient demographics.

## Introduction

Prolonged time to tracheal extubation after general anesthesia is defined as an interval of 15 minutes or longer from the recorded end of surgery to the actual removal of the tracheal tube [1-4]. This specific threshold was established to evaluate recovery profiles and value intangible costs in operating room management [1,4]. The 15-minute dichotomization is statistically and operationally valid because it exceeds the mean extubation times of all individual anesthesia groups and surgeons, ensuring that normal, brief variations were not misclassified as prolonged recovery [1,3,4]. Operationally, intervals shorter than 15 minutes are generally periods of parallel processing during which non-anesthesia personnel complete mandatory concurrent tasks such as computer charting [2]. While short intervals between the end of surgery and tracheal extubation do not translate into operating room exit delays, by the 15-minute mark, the parallel tasks are nearly always completed [2]. Thereafter, the operating room team becomes idle, transforming the delay into a direct system bottleneck [1-5]. Prolonged extubations have a mean 13.3 min longer time from the end of surgery to operating room exit (95% confidence interval 12.8-13.7 min, *p* < 0.0001), leading to overtime costs (if clinicians are paid extra for working past the end of the regular workday) or workplace dissatisfaction (if clinicians unexpectedly have to work late). At the University of Iowa, those extra minutes were beyond an eight-hour workday for 77% of prolonged extubations (73%-81%, *p* < 0.0001) [5]. The incidence of prolonged extubations among non-prone patients was 22.9% (41,768/182,374) at the University of Iowa Medical Center in the United States, with patients receiving mostly sevoflurane, and 20.5% (60/292) at Jimma Medical Center in Ethiopia, with patients receiving mostly halothane [5,6].

In our earlier cohort study of patients undergoing inhalational anesthesia, the overwhelming majority of prolonged times to tracheal extubation were caused by excessive minimum alveolar concentration (MAC) fraction doses at the completion of the surgical procedure [7]. The MAC fraction is defined as the end-tidal inhalational agent concentration divided by the agent’s altitude- and age-adjusted minimum alveolar concentration, providing a standardized, real-time measure of anesthetic depth relative to a population baseline [7-9]. Altitude adjustment addresses the fact that MAC is normalized to atmospheric pressure at sea level, which decreases with altitude. Thus, a higher concentration is needed to achieve the same effect. Age adjustment is essential because volatile anesthetic requirements decline markedly with increasing age, meaning that a fixed percentage concentration represents a much higher effective dose in an elderly patient than in a young adult [7,9-11]. Having a MAC fraction greater than 0.4 at the end of surgery is a strong predictor of prolonged extubation, with an odds ratio of 2.66 [7]. A slightly higher value, 0.5 MAC, was the threshold for intraoperative alerts in the Michigan Awareness Control Study [12]. Thus, a suitable strategy for prompt emergence is to decrease the MAC fraction to 0.5 when the degree of surgical stimulation is reduced (e.g., at the start of closing) and to maintain it as close as possible to 0.5 until surgery is complete [13,14]. Many prolonged extubations are caused by anesthesia clinicians’ planned and observed inhalational management differing markedly from this strategy [7,15]. Clinicians exhibited large case-to-case variability in dosing behaviors during surgical closure and universal difficulty in dynamically forecasting the exact timing of surgical closure, leading to insufficient timing of the reduction in agent delivery before drapes are removed [7,13-15]. 

Efforts to prevent prolonged extubations depend on clinicians relying on the MAC fraction and choosing 0.5 as the goal for the end of surgery [7-15]. The altitude adjustment is straightforward and remains constant at hospitals, but adult patients have widely varying ages. Potentially, anesthesia clinicians frequently do not fully rely on or trust population pharmacodynamic calculations displayed on modern anesthesia workstations. Earlier, Ni et al. reported MAC fractions during maintenance of anesthesia in a cohort of adult patients at Duke University, aged > 30 years [10]. They found that older patients received lower end-tidal agent concentrations, but the reduction was less than proportional to age, such that increases in age were associated with larger MAC fractions (Spearman's rank correlation of 0.12). However, their earlier study was conducted using machines that lacked a display of the age-adjusted MAC [10]. In the current study, we used data from our previously reported cohort of cases, performed with machines displaying age-adjusted MAC [7]. We tested three hypotheses.

Hypothesis 1

Our first hypothesis was that either a) there would be no association between patient age and the maintenance MAC fraction, or b) there would be a significant positive association, as in Ni et al. [10], but smaller than their observed rank correlation of 0.12.

Hypothesis 2

Our second hypothesis was that, with the MAC fraction displayed, there would be no significant residual correlation between MAC fraction and patient age at the end of the case, before tracheal extubation.

Hypothesis 3

Ourthird hypothesis was a corollary, specifically that patient age would have no association with the incidence of prolonged extubation, only the MAC fraction.

## Materials and methods

This retrospective cohort study used a subset of the same institutional dataset as our earlier investigation of inhalational agent dosing behaviors [7]. The University of Iowa Institutional Review Board determined that project #202308284 was not human subjects research, thereby waiving the requirement for written informed consent. Data were extracted from the electronic health record and the anesthesia information management system [7].

For completeness, we repeat here the details of the baseline exclusions reported in our previous article [7]. There was an initial cohort of 144,589 cases with general anesthesia, tracheal intubation and extubation in the operating room, and the absence of prone positioning [7]. Patients excluded from the older and current study were the 30,296 cases that utilized nitrous oxide and the 10,620 cases with a mean MAC fraction ≤ 0.6 for maintenance, leaving 103,673 cases [7]. The MAC fraction had to be at least 0.6 because otherwise the patients were not receiving a principally inhalational anesthetic, 0.5 being the minimum threshold simply to prevent recall [12]. Because the primary objective of our current study was to compare our data with those reported by Ni et al. [10], we further reduced the 103,673 cases by excluding 28,623 cases with patient age ≤30 years, yielding a cohort of *n* = 75,050 cases (Table 1). For all our analyses of the retrospective cohort, we used patient ages that had been calculated from birth dates in the electronic health record, not manually entered ages.

**Table 1:** Characteristics of the studied cases The data are listed in the same order as the variables in the first table of our earlier study [7]. This study cohort is limited to patients > 30 years old to match the demographic inclusion criteria of Ni et al. [10]. “MAC fraction” refers to the altitude- and age-adjusted minimum alveolar concentration as a fraction of the value for a 40-year-old [8,9]. The mean “throughout the case” was from the surgical incision through the end of surgery [7]. The sum of the counts for the three inhalational agents exceeds the total cases (103.9%) because a subset of patients received one agent for part of the case and another for the remainder.

Case or patient characteristic	% of 75,050 cases	Number of cases
Trainee, principally an anesthesia resident or student registered nurse anesthetist	47.8%	35,861
Anesthesia practitioner finished fewer than five cases with the surgeon in the past three years	80.0%	60,065
Sevoflurane mean MAC fraction throughout case ≥ 0.6	87.1%	65,385
Desflurane mean MAC fraction throughout case ≥ 0.6	4.2%	3,188
Isoflurane mean MAC fraction throughout case ≥ 0.6	12.5%	9,414
Prolonged extubation, ≥ 15 minutes from end of surgery	20.8%	15,642
American Society of Anesthesiologists’ base units ≥11	12.1%	9,095
Time from operating room entrance to the end of surgery was at least four hours	26.4%	19,800
BIS monitor used	3.1%	2,362
Age < 40 years, the age limit for adjustment of MAC fraction	13.4%	10,016
Neostigmine used	33.3%	24,965
Sugammadex used	29.0%	21,729
Succinylcholine or cisatracurium with spontaneous recovery	29.6%	22,205
Adult general surgery	24.4%	18,335
Orthopedics	18.3%	13,725
Gynecology	12.3%	9,197
Neurosurgery	10.8%	8,107
Otolaryngology	8.3%	6,254
Urology	6.8%	5,085
Ophthalmology	6.8%	5,134

For analyses of the incidence of prolonged extubation, we limited our consideration to patients within a clinically appropriate range for emergence at the end of surgery [16]. Based on established pharmacodynamic thresholds, we excluded cases if the age-adjusted MAC fraction at the end of surgery was < 0.2, the estimated MAC for amnesia in half of patients [16], or if the MAC fraction was > 1.0, the dose preventing movement in half of patients [8]. The end of surgery in the dataset was defined as the time at which the dressing was applied, when applicable [7]. We reexamined our previous graph of the unadjusted association between the percentage incidence of prolonged times to tracheal extubation and the age-adjusted MAC fraction at the end of surgery [7], limited to the same patient population as Ni et al.: patients aged > 30 years [10].

Statistical analyses were performed using Stata v19.5 (StataCorp, College Station, TX). The associations between patient age and MAC fractions were evaluated using Spearman's rank correlations, the same method as Ni et al. for direct comparison of results [10]. Again, for matching Ni et al., 95% confidence intervals were used, calculated using Fisher’s transformation and back-transformed to the correlation scale. However, consistent with our earlier article, we considered *p* < 0.01 statistically significant because the sample size was based on available data rather than power analyses for the hypotheses; see the discussion in [7]. To calculate a *p*-value comparing our observed Spearman's rank correlation coefficient with that reported by Ni et al. (0.12, with *n* = 4699) [10], we used the z-test after Fisher transformation. All reported *p*-values are two-sided. For the graphical representation of prolonged extubations, data were partitioned into 10 equally sized quantiles (deciles) based on patients’ ages and then by the MAC fraction at the end of surgery. The incidences of prolonged extubation within these deciles were presented with exact 99% binomial confidence intervals. To compare the age of patients who experienced a prolonged extubation versus those who did not, we used the two-sample Wilcoxon rank-sum (Mann-Whitney) test.

## Results

**Figure 1:**
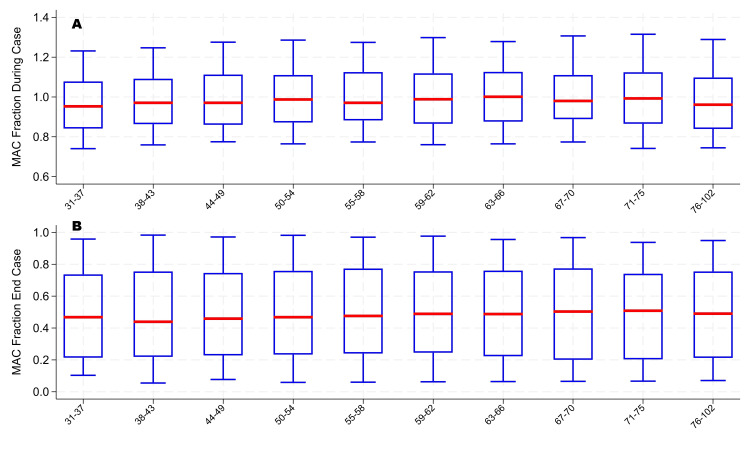
Age-adjusted MAC fractions during and at the end of surgery across patient age groups The top pane A displays the mean age-adjusted minimum alveolar concentration (MAC) fraction maintained during the surgical case, while the bottom pane B displays the age-adjusted MAC fraction at the end of surgery. The red horizontal lines represent the median (50th percentile), the blue boxes represent the interquartile range (25th to 75th percentiles), and the whiskers represent the 10th and 90th percentiles. There was a very small positive correlation between patient age and the MAC fraction during the case (Spearman's rank correlation of 0.021; *p* < 0.0001) but not at the end of surgery (rank correlation of 0.006; *p* = 0.105). For both panes, *n* = 75,050 cases.

Among *n* = 75,050 cases with care provided using anesthesia machines displaying the MAC fraction, there was a positive Spearman’s rank correlation between patient age and MAC fraction (*p* < 0.0001), but it was significantly smaller than 0.12 (*p* < 0.0001): a rank correlation of 0.021, with a 95% confidence interval of 0.014 to 0.028. The association was too small to be perceptible in pane A of Figure 1. There was no detected association between age and the MAC fraction at the end of surgery, as shown by Spearman's rank correlation of 0.006 (95% confidence interval -0.001 to 0.013; *p* = 0.105) and pane B of Figure 1.

**Figure 2:**
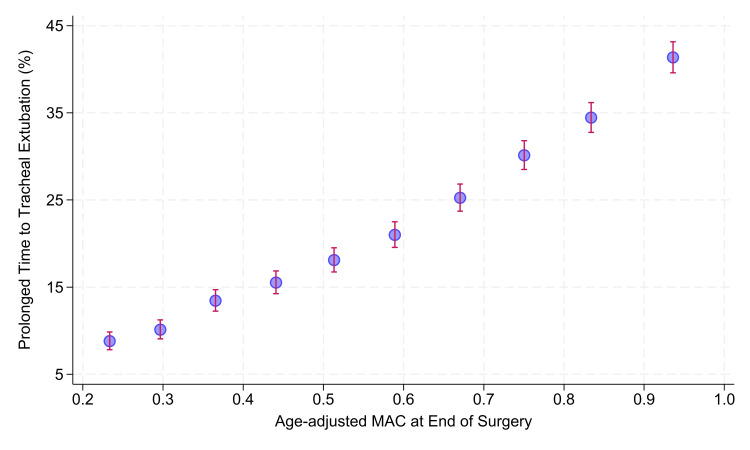
Incidence of prolonged time to tracheal extubation relative to the age-adjusted MAC fraction at the end of surgery The scatter plot demonstrates the unadjusted univariate association between the age-adjusted minimum alveolar concentration (MAC) fraction at the end of the surgical case and the incidence of prolonged tracheal extubation (≥ 15 minutes) [1-4]. Patients were divided into 10 equally sized quantiles based on their end-of-surgery MAC fraction [7]. Data points represent the mean MAC fraction for each decile. Error bars indicate the exact 99% binomial confidence intervals for the proportion of prolonged extubations [7]. To isolate the clinically relevant emergence phase, cases were excluded if the MAC fraction at the end of surgery was < 0.2, the estimated MAC for amnesia in half of patients [16], or if the MAC fraction was > 1.0, the dose preventing movement in half of patients [8]. The sample size for this figure is *n* = 51,976 cases.

Figure 2 shows a monotonically increasing (rank) unadjusted association between the percentage incidence of prolonged times to tracheal extubation and the age-adjusted MAC fraction at the end of surgery when limited to patients aged > 30 years.

**Figure 3:**
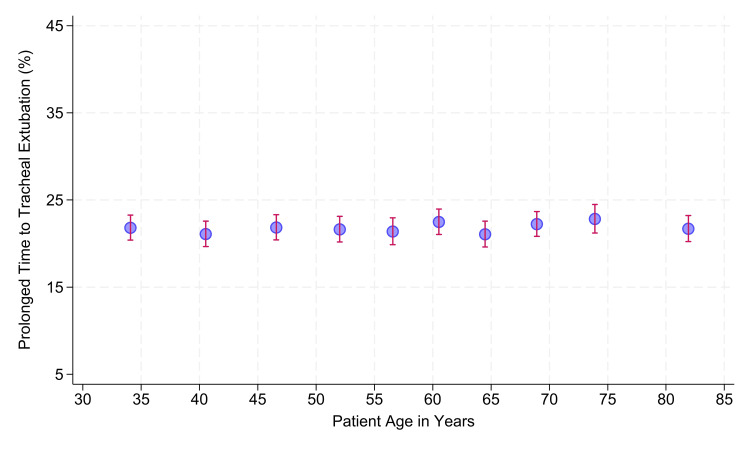
Incidence of prolonged time to tracheal extubation relative to patient age The scatter plot displays the incidence of prolonged tracheal extubation (≥ 15 minutes) as a function of patients’ ages (years). Patients were grouped into 10 equally sized age quantiles. Data points represent the mean age for each decile. Error bars indicate the exact 99% binomial confidence intervals for the proportion of prolonged extubations within each age group. Consistent with Figure 2, cases were excluded if the minimum alveolar concentration (MAC) fraction at the end of surgery was < 0.2, the estimated MAC for amnesia in half of patients [16], or if the MAC fraction was > 1.0, the dose preventing movement in half of patients [8]. The sample size for this figure is *n* = 51,976 cases.

Figure 3 shows no association between the incidence of prolonged extubation and patient age. Specifically, there was no significant association in age between patients with and without prolonged extubation (Wilcoxon rank-sum test, *p* = 0.28). The probability that a patient with prolonged extubation was older than a patient without prolonged extubation was essentially equivalent to chance (area under the receiver operating characteristic curve of 0.503).

## Discussion

As summarized in the Introduction, prolonged extubations are an economically important problem, substantially reducing operating room workflow and efficiency [1-5]. As shown in our earlier studies, most prolonged extubations are not caused by patient factors but rather by anesthesia clinicians’ dosing decisions (Figure 2) [4,5,7]. Preventing prolonged extubations will meaningfully reduce the hours anesthesiologists and nurse anesthetists work late [1-5,17]. Our collective results in Figures 1-3 show that the prolonged extubations from higher MAC fractions (> 0.5) at the end of surgery were not attributable to biased age adjustment of end-tidal agent concentrations for patient age (e.g., by mentally adjusting for age). Ni et al. observed such an association for patients receiving care when the MAC fraction was not provided, with lower end-tidal percentage concentrations, but not low enough such that older patients had higher MAC fractions [10]. We observed the same positive association for maintenance (*p* < 0.0001), but with a tiny magnitude (Spearman's rank correlation of 0.021), and fully resolved by the end of surgery (rank correlation of 0.006 and *p* = 0.105), such that age had no contribution to prolonged extubations (*p* = 0.28).

Based on our results, we have the following recommendations for anesthesiologists and nurse anesthetists [13]. Closure times have large proportional variability, even greater than surgical times [18]. Times remaining in surgical cases do not decrease like simple countdown timers [18-20]. Therefore, if using an anesthesia machine with end-tidal control, reduce the MAC fraction to 0.5 as soon as major stimulation has ended. If agent levels need to be adjusted manually, as at the University of Iowa, turning the vaporizer off and fresh gas flows up poses the risk of forgetting to turn the vaporizer back on [21] if distracted, and the closure takes longer than expected. Instead, turn the vaporizer down to the age-adjusted MAC-awake (e.g., 0.62% sevoflurane in a 40-year-old [22]) and increase the fresh gas flow up to 8.0 liters per minute [13]. When a 0.5 MAC fraction is achieved, maintain that level until surgery is done by titrating the vaporizer and reducing the fresh gas flow (e.g., to 1.0 L/min) [13].

On 18 June 2026, we updated our literature review for other related reports. Using PubMed, (prolonged extubation*[TIAB] OR delayed extubation*[TIAB]) AND ("minimum alveolar"[TIAB] OR "MAC"[TIAB]), the three results were Dexter et al. (2024), Clevenger et al. (2024), and a related article by the current investigators that included examination of the use of the BIS monitor [7,15,23]. There was no association between prolonged extubation and ≥100 minutes of BIS monitor use (29.3% without BIS monitor versus 29.5% with BIS monitor; both with standard error < 0.5%) [23]. Ni et al. also considered the BIS, but on a topic different than the current article, specifically BIS for maintenance of anesthesia and patient age [10]. Across many patients, lower MAC fractions are, of course, associated with higher BIS [24,25]. However, when comparing within patients, the associations are weak and often undetected [24,25]. The age-adjustment of MAC differs proportionately from changes in BIS with patient age [26]. We are unaware of any study suggesting that dynamic titration of inhalational anesthetics based on BIS improves wake-up timeliness when controlling for the associated MAC fraction.

A strength of our article was that all our analyses used the actual patient ages, regardless of the ages entered by the anesthesia clinicians [7,27]. Specifically, the patient age deciles on the horizontal axes of Figures 1-3 are the true patient ages. The MAC fractions on the vertical axes of Figure 1 were calculated from the end-tidal agent measurements and true patient ages. Any potential data-entry errors or rounding of patient ages made by clinicians when entering data into the anesthesia machine would contribute to the quantified statistical variability (e.g., as reflected in the error bars in the figures) but would not bias our results. A limitation in understanding clinicians' behavior is that we do not know how often the age was entered (i.e., the MAC fraction displayed was age-adjusted rather than reported using the default age of 40 years) [7,27]. However, Figure 1B and Figure 3 show that the limitation did not affect the conclusions for the population, those of interest, given that there were at most negligible residual effects of patient age. A follow-up study can evaluate whether the statistically significant (p < 0.0001) but tiny residual Spearman's correlation of 0.021 reverts to zero when the patient’s age is known to be used.

Regarding additional study limitations, prolonged times to tracheal extubation rarely occur with nitrous oxide or desflurane alone [1,4]. However, both agents are rarely used anymore in many countries. Second, our analyses of prolonged extubations were limited to cases that ended with an age-adjusted MAC fraction between 0.2 and 1.0. Our conclusions do not apply to the age adjustment of intravenous anesthetics or to deep extubations. Third, our single-center study was limited to patients aged > 30 years to match Ni et al. [10]. Finally, our sample size was based on available electronic health record data through the time when anesthesia machines were changed [7]. However, our confidence intervals were very narrow, suggesting that there were enough cases to detect any important residual association between the MAC fraction at the end of surgery and age.

## Conclusions

Prolonged times to tracheal extubation lead to substantial reductions in operating room workflow and anesthesia clinicians’ productivity. Our findings demonstrate that when using anesthesia workstations equipped with real-time, age-adjusted minimum alveolar concentration (MAC) fraction displays, the confounding effect of patient age on anesthetic maintenance dosing is effectively neutralized. At the completion of surgery, there is no residual correlation between patient age and the remaining depth of anesthesia. Consequently, the incidence of prolonged extubation is independent of patient age, confirming that delayed emergence is driven by clinician titration behaviors rather than patient demographics.
